# Designing for dissemination among public health and clinical practitioners in the USA

**DOI:** 10.1017/cts.2023.695

**Published:** 2023-12-14

**Authors:** Thembekile Shato, Maura M. Kepper, Gabriella M. McLoughlin, Rachel G. Tabak, Russell E. Glasgow, Ross C. Brownson

**Affiliations:** 1 Prevention Research Center, Brown School, Washington University in St. Louis, St. Louis, MO, USA; 2 Department of Surgery (Division of Public Health Sciences), Washington University School of Medicine, Washington University in St. Louis, St. Louis, MO, USA; 3 College of Public Health, Temple University, Philadelphia, PA, USA; 4 Implementation Science Center for Cancer Control, Brown School and School of Medicine, Washington University in St. Louis, St. Louis, MO, USA; 5 Department of Family Medicine and ACCORDS Research Center, University of Colorado Anschutz Medical Campus, Aurora, CO, USA

**Keywords:** Dissemination research, implementation science, public health practice, primary health care, evidence-based health practice

## Abstract

**Introduction:**

The slow adoption of evidence-based interventions reflects gaps in effective dissemination of research evidence. Existing studies examining designing for dissemination (D4D), a process that ensures interventions and implementation strategies consider adopters’ contexts, have focused primarily on researchers, with limited perspectives of practitioners. To address these gaps, this study examined D4D practice among public health and clinical practitioners in the USA.

**Methods:**

We conducted a cross-sectional study among public health and primary care practitioners in April to June 2022 (analyzed in July 2022 to December 2022). Both groups were recruited through national-level rosters. The survey was informed by previous D4D studies and pretested using cognitive interviewing.

**Results:**

Among 577 respondents, 45% were public health and 55% primary care practitioners, with an overall survey response rate of 5.5%. The most commonly ranked sources of research evidence were email announcements for public health practitioners (43.7%) and reading academic journals for clinical practitioners (37.9%). Practitioners used research findings to promote health equity (67%) and evaluate programs/services (66%). A higher proportion of clinical compared to public health practitioners strongly agreed/agreed that within their work setting they had adequate financial resources (36% vs. 23%, *p* < 0.001) and adequate staffing (36% vs. 24%, *p* = 0.001) to implement research findings. Only 20% of all practitioners reported having a designated individual or team responsible for finding and disseminating research evidence.

**Conclusions:**

Addressing both individual and modifiable barriers, including organizational capacity to access and use research evidence, may better align the efforts of researchers with priorities and resources of practitioners.

## Introduction

Although there has been significant investment in health-related research and development of interventions, translation into policy and routine practice remains slow [1,2]. For example, in national studies among US public health departments, an estimated 58%–64% of programs and policies were reported as evidence-based [3]. In another study among public health practitioners in health departments, an estimated three-quarters (75%) of programs were reported as evidence-based in two US states [6]. Ebell and colleagues also found that 51% of clinical recommendations for primary care practice were based on patient-oriented evidence from original research, with only 18% based on high-quality evidence [7]. These studies reflect gaps and barriers and suggest that evidence-based interventions (EBIs) are not being disseminated effectively [1,8–10].

The lingering research to practice gap is attributed to interacting barriers at multiple levels shaped by political, economic, cultural, scientific, and organizational contexts [11]. These barriers include the lack of relevance of research findings to practice, research findings not packaged for ease of implementation, limited capacity and resources to disseminate or apply research, lack of organizational and structural supports to enhance access and adoption of research, and lack of funding [11–13].

Dissemination, an active and intentional process of spreading EBIs to target audiences via determined channels using planned strategies [14], is a critical step for effective adoption and implementation of these EBIs [15]. The process of dissemination is influenced by multiple factors related to characteristics of the individual, innovation, organization, and environment [16]. However, previous studies have shown that dissemination is too often passive and not aligned between those producing (often researchers) and those applying the research evidence (often practitioners) [17–19], contributing to low uptake of EBIs [20]. Dissemination approaches by researchers typically include publication in journals and presentations at conferences. Although such practices are important and effective for other researchers, they do not line up well with needs and communications approaches and preferences of practitioners who are the target adopters and implementers of research evidence [17]. Designing for dissemination (D4D) seeks to address this disconnect to better align how researchers produce and communicate research evidence (push) with how practitioners or policymakers receive and utilize (pull) research evidence, and the structural supports needed to support evidence-based practice (capacity) [18]. D4D is a process to ensure that products of research are designed and developed to match the contextual characteristics (i.e., needs, assets, and resources) of the target audiences, including practitioners, and their setting [14,18,21].

Previous studies have examined the practice of D4D primarily among researchers. In a study among researchers in academic and national research institutions in the USA found that 73% spent less than 10% of their time on dissemination, 53% had a person or team in their unit dedicated to dissemination, and only a third (34%) involved stakeholders in the process [8]. A more recent study among dissemination and implementation researchers in the USA and Canada found that overall engagement in dissemination-related activities and stakeholder involvement in the research were more common [22]. However, dissemination-related activities (i.e., face-to-face meetings) identified as most impactful to practice or policy were used by only 40% of respondents, while dissemination-related activities such as journal publications, conference presentations, and reports to funders were used by the majority (>70%) of respondents [22]. Organizational structures and supports, including dissemination expected by funding agencies and previous work in a practice or policy setting, were identified as significant and most important determinants of dissemination efforts by public health researchers to non-research audiences [13].

Most prior D4D studies focused mostly on researchers (the push side), with few studies examining the perspectives of practitioners (the pull side). A qualitative study that explored the use of research evidence among public health officials in the USA found that most respondents used research to support grant writing; and primary sources of research evidence were professional organizations and government agencies, compared to research journals [23]. In this same study, respondents also indicated a desire to participate in the planning phase of research projects and recommended simplifying for and tailoring for diverse target audiences to enhance usefulness of research evidence [23]. Previous studies in Canada found that public health decision-makers in public health departments and community organizations preferred executive summaries of research evidence [24,25]. Additionally, organizational characteristics including perceived organizational value on use of research evidence and ongoing training were shown to be influential in the use of research evidence [26]. The divergence in perspectives between researchers and practitioners is also reflected in training programs that have focused primarily on building capacity for implementation in researchers [3,19], and Kwan and colleagues stressed that dissemination strategies have focused much more on the push side (researchers’ perspective) with little emphasis on the pull side (practitioners’ perspective), warranting more emphasis on practitioner engagement in dissemination [18]. Since the COVID-19 pandemic, dissemination practices may have changed and thus we need to understand practitioners’ perspectives and preferences to expand our understanding of D4D.

By ensuring that research and interventions are designed and developed in ways that match with priorities and needs of adopters and implementers, D4D approach has the potential to improve the translation of evidence into practice [8,18]. D4D provides the avenue to identify all key stakeholders and collaboratively develop dissemination and implementation approaches that reflect the experiences of adopters, implementers, and beneficiaries of research evidence – a critical step toward achieving health equity [18,27]. Thus, this study aims to examine and describe the practice and patterns of D4D among practitioners in the USA. Information from this study will guide the co-design of dissemination products (e.g., research findings and interventions) and strategies engaging relevant stakeholders to maximize the reach and adoption of research evidence and EBIs.

## Methods

### Study Design and Participants

This cross-sectional study was conducted across the USA among public health and clinical practitioners in spring 2022. Public health practitioners were considered those working in local and state health departments. Clinical practitioners were primary care physicians working in the following settings: pediatrics, family medicine, internal medicine, obstetrics and gynecology, and emergency medicine.

### Survey Development and Measures

The survey development was informed by three theoretical frameworks, including Diffusion of Innovations, Knowledge to Action (K2A) Frameworks, and Reach Effectiveness Adoption Implementation and Maintenance (RE-AIM) as well as previous D4D studies [8,22,28–30]. Diffusion of Innovations helps to understand the spread of new or innovative ideas (e.g., research evidence) and characteristics of adopters, proposing that adoption of an innovation is accomplished in several stages beginning with the awareness of the innovation to the continued use of the innovation [31,32]. The K2A framework highlights the key elements and outcomes of knowledge (e.g., research evidence) utilization in practice including the need to identify and understand multi-level factors (barriers and facilitators) that influence knowledge use. RE-AIM focuses on implementation outcomes and dimensions that together determine the public health impact of a program or policy (e.g., reach and implementation) [33–36], The linkage between survey content and these theoretical frameworks is summarized in Supplementary material 1. The survey was pretested by conducting cognitive interviewing using the think-aloud technique [37,38] among 10 public health practitioners (*n* = 5) and clinical practitioners (*n* = 5). The practitioners who pretested the survey were recruited through the professional networks within the Prevention Research Center at Washington University in St Louis. Responses from the interviews informed the revision of the survey, including re-wording and addition or deletion of survey questions to enhance relevance and readability of the survey. The final survey had 24 questions (see Supplementary material 2).

The survey assessed individual (practitioner) and organizational factors. First, awareness and knowledge of research evidence (e.g., Diffusion of Innovation [31,32]) included items assessing sources of information about and characteristics of presenting research evidence as well as whether an individual or team was designated in the organization or clinic to find and report research evidence. Second, adoption and implementation of research evidence (K2A [33,34] and RE-AIM [35,36]) included items assessing the frequency, barriers, and facilitators of using research evidence. For the final section, engagement in research included items addressing ways in which respondents were involved in research within the past 2 years, including the impact of the COVID-19 pandemic.

### Data Collection

Practitioners were recruited online through local- and national-level rosters – Missouri local health departments for the local public health practitioners, National Association of Chronic Disease Directors (NACDD) for the state health public health practitioners, and American Medical Association (AMA) for the clinical practitioners. Surveys were self-administered and conducted online through Qualtrics (Qualtrics, 2020). An initial email invitation in addition to three email reminders with a unique link to the survey were sent to a random sample of respondents from each list. Data were collected from April to June 2022. Upon completion of the survey, participants received a $50 gift card. This study was approved by Washington University in St Louis Institutional Review Board (IRB No. 202112167).

### Analysis

Descriptive analyses were conducted to summarize data using frequencies (percentages) for categorical variables and means (standard deviations) for continuous variables. Subgroup analyses exploring differences between public health and clinical practitioners were assessed in bivariate analyses using chi-squared tests. All analyses were conducted in SAS v9.4.

## Results

After deleting 44 invalid responses (e.g., duplicates), analysis was conducted on 623 respondents. The final analytic sample (*n* = 577) excluded 45 respondents who considered themselves only as researchers and 1 respondent who indicated they had retired. The overall response rate for the survey was 5.5% [9.1% (*n* = 41/451) among local public health practitioners, 22.5% (*n* = 262/1,162) among state public health practitioners, and 3.3% (320/9,648) among clinical public health practitioners]. Among 577 respondents, 55% were clinical practitioners and 45% were public health practitioners (Table 1). State public health practitioners (*n* = 222, 85%) comprised the majority of public health practitioners. The highest proportion of public health practitioners (81%) worked in state health departments, and the highest proportion of clinical practitioners (64.8%) worked in outpatient health facilities. The majority of clinical practitioners (95.7%) had a doctoral degree and public health practitioners had a master’s degree (58%). One-fifth of the respondents considered themselves as both practitioners and researchers (19%). The highest proportion of all practitioners ranked national government agencies (40%), followed by professional associations (27%) and researchers (21%) as their most common source of information for research findings (Table 2). All practitioners most commonly trusted email announcements (31%), reading academic journals (27%), and professional conferences (10%) as their source of information. For public health practitioners, the most common trusted sources of information were national government agencies (55%) followed by researchers (21%), and most often got information about research findings from email announcements (44%) followed by government reports (14%) and reading academic journals (13%). For clinical practitioners, the most trusted sources of information were professional associations (37%) and national government agencies (28%), and the most commonly ranked source of research findings was reading academic journals (38%) followed by email announcements (20%) and professional conferences (13%).

**Table 1.** Characteristics of survey respondents (n = 577)

Demographic characteristics	All (*n* = 577) *n* (%)	Public health practitioner (*n* = 261) *n* (%)	Clinical practitioner (*n* = 316) *n* (%)
**Primary work setting (*n* = 575)**			
Healthcare facility – inpatient (e.g., hospital, clinic)	52 (9.0)	0 (0.0)	52 (16.5)
Healthcare facility – outpatient (e.g., hospital, clinic)	204 (35.5)	0 (0.0)	204 (64.8)
Local health department	46 (8.0)	43 (16.5)	3 (1.0)
State health department	214 (37.2)	211 (81.2)	3 (1.0)
University or school	14 (2.4)	1 (0.4)	13 (4.1)
Community-based organization	20 (3.5)	1 (0.4)	19 (6.0)
National organization^1^	3 (0.5)	3 (1.2)	0 (0.0)
Other^2^	22 (3.8)	1 (0.4)	21 (6.7)
**Education (*n* = 575)**			
High school graduate^3^	1 (0.2)	1 (0.4)	0 (0.0)
Some college	5 (0.9)	4 (1.5)	1 (0.3)
Bachelor’s degree	55 (9.6)	51 (19.6)	4 (1.3)
Master’s degree	158 (27.3)	150 (57.7)	8 (2.5)
Doctoral degree (e.g., MD, PhD)	347 (60.4)	46 (17.7)	301 (95.6)
Professional degree (e.g., LPN)	5 (0.9)	4 (1.5)	1 (0.3)
Other	5 (0.9)	4 (1.5)	0 (0.0)
**Academic areas of formal degree**			
Medicine	314 (54.4)	14 (5.4)	300 (94.9)
Public health	167 (28.9)	151 (57.9)	16 (5.1)
Other^4^	83 (14.4)	72 (27.6)	11 (3.5)
Behavioral Science	40 (6.9)	37 (14.2)	3 (1.0)
Natural Sciences	37 (6.4)	15 (5.8)	22 (7.0)
Nursing	34 (5.9)	26 (10.0)	8 (2.5)
Policy	17 (3.0)	15 (5.8)	2 (0.6)
Health services research	13 (2.3)	7 (2.7)	6 (1.9)
**Highest degree year (*n* = 574)**			
1955–1990	123 (21.4)	35 (13.5)	88 (28.0)
1991–2000	174 (30.3)	57 (21.9)	117 (37.3)
2001–2010	154 (26.8)	72 (27.7)	82 (26.1)
2010–2022	123 (21.4)	96 (36. 9)	27 (8.6)
**Consider myself as: (*n* = 573)**			
Both Practitioner and Researcher	111 (19.4)	70 (27.1)	41 (13.0)
Practitioner	462 (80.6)	188 (72.9)	274 (87.0)
**Region (*n* = 568)**			
Midwest	158 (27.8)	76 (29.5)	82 (26.5)
West	133 (23.4)	76 (29.5)	57 (18.4)
Northeast	126 (22.2)	41 (15.9)	85 (27.4)
Southeast	105 (18.5)	53 (20.5)	52 (16.8)
Southwest	41 (7.2)	8 (3.1)	33 (10.7)
Other^5^	5 (0.9)	4 (1.6)	1 (0.3)

1 Ministry of Health.

2 Private practice, locum, VA, Telemedicine, Corporation.

3 Trade/technical/vocational education beyond high school.

4 Includes Nutrition/Dietetics, Dentistry, Public Administration/Social Welfare, Education, History, Law, Economics.

5 Virgin Islands, Micronesia, Palau.

**Table 2.** Information sources for research findings (n = 577)

	Public health practitioners (*n* = 261)			Clinical practitioners (*n* = 316)		
	Rank 1^st^ *n* (%)	Ranked in top 3 *n* (%)	Rank for #1^3^	Rank 1^st^ *n* (%)	Ranked in top 3 *n* (%)	Rank for #1^3^
**Most often get information**						
Email announcements	114 (43.7)	163 (62.5)	1	63 (19.9)	151 (47.8)	2
Reading academic journals	34 (13.0)	98 (37.6)	3	120 (38.0)	253 (80.1)	1
Professional conferences	19 (7.3)	100 (38.3)	5	40 (12.7)	187 (59.2)	3
Government reports	36 (13.8)	105 (40.2)	2	13 (4.1)	65 (20.6)	5
Newsletters	11 (4.2)	67 (25.7)	6	19 (6.0)	75 (23.7)	4
Webinars	20 (7.6)	107 (41.0)	4	8 (2.5)	53 (16.8)	8
Face to face/virtual meetings with stakeholders	13 (5.0)	46 (17.6)	7	14 (4.4)	38 (12.0)	5
Policy briefs	10 (3.8)	63 (24.1)	8	5 (1.6)	24 (7.6)	10
Podcasts	0 (0.0)	6 (2.3)	11	11 (3.5)	28 (8.9)	7
Social media	2 (0.8)	14 (5.4)	9	6 (1.9)	18 (5.7)	9
Other^1^	0 (0.0)	7 (2.7)	10	5 (1.6)	13 (4.1)	10
**Most trusted sources of information**						
National government agencies	144 (55.2)	244 (93.5)	1	88 (27.9)	263 (83.2)	2
Professional associations	39 (14.9)	208 (79.7)	3	116 (36.7)	253 (80.1)	1
Researchers	55 (21.1)	138 (52.9)	2	66 (20.9)	173 (54.8)	3
State government agencies	8 (3.1)	116 (44.4)	4	14 (4.4)	99 (31.3)	4
Other^2^	3 (0.9)	6 (2.3)	6	11 (3.5)	20 (6.3)	5
Local government agencies	6 (2.3)	30 (11.5)	5	3 (1.0)	50 (15.8)	6
Advocacy organizations	1 (0.4)	26 (9.6)	7	3 (1.0)	16 (5.1)	6
Social media	1 (0.4)	2 (0.8)	7	1 (0.3)	4 (1.3)	8
News media	0 (0.0)	4 (1.5)	9	1 (0.3)	15 (4.8)	8

1 Includes internet search; news media; Up to Date website.

2 Includes academic journals; Pubmed; Up to Date website.

3 Top ranked source of information in ascending order (from most common to least common).

Overall, the majority of all practitioners reported that when presenting research findings, it is very or extremely important that information be relevant to the patients or populations served (92%), provides practical advice about implementation (89%), tells a story of how patients or populations served are affected by an issue (69%), provides data on cost-effectiveness (57%), and is delivered by someone known and respected (50%) (Table 3). Compared to clinical practitioners, a significantly higher proportion of public health practitioners indicated that it was extremely or very important for research findings to be relevant to the populations served (95% vs. 88%, *p* = 0.005), to present practical advice about implementation (95% vs. 84%, *p* = 0.001), and tell a story about how an issue affects populations served (78% vs. 62%, *p* < 0.001).

**Table 3.** Important characteristics of presenting research findings

	All (*n* = 577) *n* (%)	Clinical practitioner (*n* = 316) *n* (%)	Public health practitioner (*n* = 261) *n* (%)	*P*-value^1^
**Relevant to the patients or populations we serve**				**0.005**
Very/extremely important	527 (91.5)	278 (88.3)	249 (95.4)	
Moderately important	45 (7.8)	34 (10.8)	11 (4.2)	
Not at all/slightly important	4 (0.7)	3 (0.9)	1 (0.4)	
**Presents practical advice about implementation**				**0.001**
Very/extremely important	511 (88.7)	264 (83.8)	247 (94.6)	
Moderately important	59 (10.2)	45 (14.3)	14 (5.4)	
Not at all/slightly important	6 (1.0)	6 (1.9)	0 (0.0)	
**Tells a story of how an issue affects the patients/populations we serve**				**0.000**
Very/extremely important	399 (69.3)	196 (62.2)	203 (77.8)	
Moderately important	125 (21.7)	82 (26.0)	43 (16.5)	
Not at all/slightly important	52 (9.0)	37 (11.8)	15 (5.8)	
**Provides data on cost-effectiveness**				0.318
Very/extremely important	328 (56.9)	185 (58.7)	143 (54.8)	
Moderately important	195 (33.9)	106 (33.7)	89 (34.1)	
Not at all/slightly important	53 (9.2)	24 (7.6)	29 (11.1)	
**Delivered by someone I know and respect**				**0.044**
Very/extremely important	289 (50.2)	171 (54.3)	118 (45.2)	
Moderately important	163 (28.3)	87 (27.6)	76 (29.1)	
Not at all/slightly important	124 (21.5)	57 (18.1)	67 (25.7)	
**Provides data on access/insurance coverage**				0.239
Very/extremely important	282 (49.0)	159 (50.5)	123 (47.1)	
Moderately important	196 (34.0)	98 (31.3)	98 (37.6)	
Not at all/slightly important	98 (17.0)	58 (18.4)	40 (15.3)	

1 Bolded *p*-value significant at *p* < 0.05, based on tests of differences between clinical and public health practitioners.

There were significant differences in the uses of research findings between public health and clinical practitioners (Table 4). Public health practitioners, compared to clinical practitioners, were more likely to every time or almost every time use research findings to promote health equity (80% vs. 56%, *p* < 0.001), evaluate programs/policies/services (83% vs. 52%, *p* < 0.001), address the spread of inaccurate information (64% vs. 57%, *p* < 0.001), modify existing programs/services (75% vs. 44%, *p* < 0.001), develop new programs/services (82% vs. 38%, *p* < 0.001), discontinue an existing program/service (45% vs. 35%, *p* < 0.001), and to write a grant application (71% vs. 13%, *p* < 0.001). Lack of time to find research was ranked as the most common barrier for both public health (44%) and clinical practitioners (37%), followed by lack of relevance of research to work needs for public health practitioners (17%), and lack of a brief summary of research findings for clinical practitioners (15%). For both public health and clinical practitioners, easy access to a summary of research findings (30% and 36%), easy access to research findings or data sources (30% vs. 26%), and leaders or direct supervisors placing high priority on research (23% and 13%) were the most important facilitators of using research findings.

**Table 4.** Uses of research findings (*n* = 577)

	All (*n* = 577) *n* (%)	Public health practitioner (*n* = 261) *n* (%)	Clinical practitioner (*n* = 316) *n* (%)	*P*-value^1^
**Promote health equity**				**<0.001**
Almost every time/every time	384 (66.6)	208 (79.7)	176 (55.7)	
Sometimes	124 (21.5)	43 (16.5)	81 (25.6)	
Never/almost never	45 (7.8)	3 (1.2)	42 (13.3)	
Not applicable/missing	24 (4.2)	7 (2.7)	17 (5.4)	
**Evaluate programs/policies/services**				**<0.001**
Almost every time/every time	381 (66.0)	217 (83.1)	164 (51.9)	
Sometimes	126 (21.8)	32 (12.3)	94 (29.8)	
Never/almost never	48 (8.3)	6 (2.3)	42 (13.3)	
Not applicable/missing	22 (3.8)	6 (2.3)	16 (5.1)	
**Address the spread of inaccurate information**				**0.000**
Almost every time/every time	347 (60.1)	166 (63.6)	181 (57.3)	
Sometimes	131 (22.7)	51 (19.5)	80 (25.3)	
Never/almost never	55 (9.5)	14 (5.4)	41 (13.0)	
Not applicable/missing	44 (7.6)	30 (11.5)	14 (4.4)	
**Modify an existing program/policy/service**				**<0.001**
Almost every time/every time	336 (58.2)	196 (75.1)	140 (44.3)	
Sometimes	174 (30.2)	55 (21.2)	119 (37.7)	
Never/almost never	47 (8.2)	5 (1.9)	42 (13.3)	
Not applicable/missing	20 (3.5)	5 (1.9)	15 (4.8)	
**Develop a new program/policy/service**				**<0.001**
Almost every time/every time	334 (57.9)	213 (81.6)	121 (38.3)	
Sometimes	151 (26.2)	37 (14.2)	114 (36.1)	
Never/almost never	66 (11.4)	4 (1.5)	62 (19.6)	
Not applicable/missing	26 (4.5)	7 (2.7)	19 (6.0)	
**Plan or conduct a needs assessment**				**<0.001**
Almost every time/every time	260 (45.1)	182 (69.7)	78 (24.7)	
Sometimes	138 (23.9)	51 (19.5)	87 (27.5)	
Never/almost never	128 (22.2)	8 (3.1)	120 (38.0)	
Not applicable/missing	51 (8.8)	20 (7.7)	31 (9.8)	
**Discontinue an existing program/policy/service**				**0.000**
Almost every time/every time	227 (39.3)	118 (45.2)	109 (34.5)	
Sometimes	208 (36.1)	86 (33.0)	122 (38.6)	
Never/almost never	89 (15.4)	26 (10.0)	63 (19.9)	
Not applicable/missing	53 (9.2)	31 (11.9)	22 (7.0)	
**Write a grant application**				**<0.001**
Almost every time/every time	226 (39.2)	185 (70.9)	41 (13.0)	
Sometimes	177 (13.3)	47 (18.0)	30 (9.5)	
Never/almost never	204 (35.4)	16 (7.8)	188 (59.5)	
Not applicable/missing	70 (12.1)	13 (5.0)	57 (18.0)	

1 Bolded *p*-value significant at *p* < 0.05, based on tests of differences between clinical and public health practitioners.

Table 5 presents organizational factors related to use of research findings. A significantly lower proportion of public health practitioners compared to clinical practitioners strongly agreed or agree they had adequate staffing to implement research findings in their work (24% vs. 36%, *p* = 0.007) and adequate financial resources to implement research findings (23% vs. 36%, *p* < 0.001). The majority of practitioners (83%) placed a priority on promoting health equity in their work and indicated that it was extremely or very important for the organization/clinic to use research findings. Overall, 20% of the respondents had a designated individual or team responsible for findings and disseminating research findings.

**Table 5.** Organizational setting and supports in using research findings (n = 577)

	All *n* (%)	Public health practitioners *n* (%)	Clinical practitioners *n* (%)	*P*-value^1^
**Organizational value**				
Importance of using research findings in the organization/clinic’s efforts (*n* = 575)				**<0.001**
Very important/extremely important	335 (58.3)	187 (71.7)	148 (47.1)	
Moderately important	165 (28.7)	61 (23.4)	104 (33.1)	
Not at all important/slightly important	75 (13.0)	13 (5.0)	63 (20.0)	
Place a priority on promoting health equity (*n* = 559)				**<0.001**
Strongly agree/agree	465 (83.2)	234 (92.9)	231 (75.2)	
Neither agree nor disagree	70 (12.5)	15 (6.0)	55 (17.9)	
Strongly disagree/disagree	24 (4.3)	3 (1.2)	21 (6.8)	
**Organizational capacity**				
Have adequate staffing to implement research findings (*n* = 568)				**0.007**
Strongly agree/agree	173 (30.5)	62 (24.0)	111 (35.8)	
Neither agree nor disagree	139 (24.5)	65 (25.2)	74 (23.9)	
Strongly disagree/disagree	256 (45.1)	131 (50.8)	125 (40.3)	
Have adequate financial resources to implement research findings (*n* = 559)				**0.000**
Strongly agree/agree	167 (29.8)	58 (22.8)	109 (35.9)	
Neither agree nor disagree	150 (26.8)	63 (24.7)	87 (28.6)	
Strongly disagree/disagree	242 (43.3)	134 (52.6)	108 (35.5)	
Designated individual or team responsible for finding and disseminating information on research findings (*n* = 575)				0.236
Yes	114 (19.8)	48 (18.4)	66 (21.0)	
No	360 (62.6)	173 (66.3)	187 (59.6)	
Not sure	101 (17.6)	40 (15.3)	61 (19.4)	
**Organizational activities**				
Adapt to incorporating research findings (*n* = 571)				0.186
Strongly agree/agree	445 (77.9)	208 (80.3)	237 (76.0)	
Neither agree nor disagree	80 (14.0)	36 (13.9)	44 (14.1)	
Strongly disagree/disagree	46 (8.1)	15 (5.8)	31 (10.0)	
Track and monitor the use of research findings (*n* = 557)				0.659
Strongly agree/agree	176 (31.6)	85 (33.5)	91 (30.0)	
Neither agree nor disagree	148 (26.6)	67 (26.4)	81 (26.7)	
Strongly disagree/disagree	233 (41.9)	102 (40.2)	131 (43.2)	

1 Bolded p-value significant at *p* < 0.05, based on tests of differences between clinical and public health practitioners.

The majority of all survey practitioners in the survey (57%) indicated that research involvement (e.g., serving on an advisory committee or as a research participant, disseminating research findings) since COVID-19 stayed the same. Overall, about a third of practitioners reported being involved in collecting data, interpreting data, and dissemination findings through personal or professional networks. In the past 2 years, a significantly higher proportion of public health practitioners were involved with collecting data (51% vs. 24%, *p* < 0.001), interpreting data (45% vs. 21%, *p* < 0.001), and disseminating findings through personal or professional networks (46% vs. 20%, *p* < 0.001) compared to clinical practitioners. A significantly higher proportion of clinical practitioners compared to public health practitioners had not been involved in research in the past 2 years (45% vs. 20%, *p* < 0.001).

## Discussion

This study addresses a critical gap in D4D by examining the perspectives of practitioners who are adopters and implementers of research. The most common source of research findings were academic journals and email announcements for clinical and public health practitioners. Email announcements could include newsletters, reports, or web links with information about research findings. The majority of all practitioners in the survey, with significantly higher proportions among public health practitioners, frequently used research findings to promote health equity, address the spread of inaccurate information, and develop, modify, and evaluate programs or services. The most common barrier to using research was a lack of time, while easy access to research evidence was the most common facilitator. Only a third of the practitioners had adequate staffing and financial resources to find and implement research in their work.

Consistent with previous findings in both public health practice and health care [11,12,23,39–41], practitioners in our study reported time constraints as the most common barrier to finding and using research in practice. For example, in a survey among state-level public health practitioners, respondents commonly cited lack of time as a barrier to using evidence-based decision-making in practice [41]. In qualitative studies among healthcare providers including pediatric surgeons and allied health clinicians, the already demanding day-to-day workload poses time constraints not only to patient care but also to prioritizing and using research evidence in practice [39,40]. As outlined in a review of factors influencing research translation to practice, many practice settings are faced with competing priorities, tasks, and demands which may exacerbate the challenge of finding and integrating research [11]. Narain and colleagues note that within a practice or policy setting, there is need to identify and take quick action on feasible solutions which may not always be appreciated or often accounted for in the process of research production and dissemination [23].

The biggest facilitator for using research evidence among practitioners in our study was easy access to research and a summary of research findings. These data are similar to findings from a qualitative study among public health officials who favored summaries and systematic reviews as a way of consuming research evidence [23,26]. This underscores the need for strategies that better align with practitioners’ preferences and simplify the access, retrieval, and integration of research evidence which may, in turn, help to overcome time constraints within practice settings.

We found major differences in research involvement, a key aspect of D4D, between practitioners and researchers in previous studies. All practitioners were more involved in research during the end stages of data collection, interpreting data, and disseminating findings through personal or professional networks. In contrast, previous D4D studies among researchers reported research engagement activities more toward the beginning of the research process. In two D4D studies among researchers in the USA and Canada, the most common methods of involvement included development of research advisory committees (66%–72%), engagement of persons with diverse experiences, perspectives and roles in research proposal development and implementation to enhance relevance of research to practice settings (62%) and to stakeholders (59%), and participation on the research team (63%) [8,22]. This suggests that there is still a need to identify ways to intentionally engage practitioners from conceptualization and throughout the research process. Engagement is critical to enhancing the relevance and translation of research to practice. Although half of practitioners indicated that their involvement in research did not change, the discrepancy in findings in our study may also reflect the challenges faced during the COVID-19 pandemic in bringing stakeholders together for research.

The second difference was related to dissemination-related activities between researchers and practitioners. We found that less than a third of all practitioners, with a much lower proportion among public health practitioners, most often got research information from reading academic journals. Yet, this is the most common approach for disseminating research evidence used by researchers [42]. Knoepke and colleagues found that dissemination and implementation researchers most frequently disseminated their work by publishing in academic journals (88%), delivering conference presentations (86%), and reporting to funders (74%) [22]. In this same study, use of dissemination-related activities most impactful to practice were used less frequently [22]. The third contrast in findings from our study compared to that of other studies related to organizational supports. Only 19.8% of practitioners reported having a designated individual or team for finding and disseminating research evidence. This is much lower than reported in a previous study where over half (53%) of the researchers had a person or team within their unit dedicated to dissemination [8]. This may reflect differences in capacity and structural supports available and accessible to practitioners compared to researchers, particularly those working in institutions with a significant focus on research, such as reported in the 2018 survey study of public health researchers [8].

Ours is one of several recent findings highlighting the persistent push–pull disconnect between researchers and practitioners [18,42]. which has implications for the translation and integration of research evidence into practice or policy [23]. To improve the translation of research into practice, there is an urgent need to better align and match approaches, preferences, and priorities of researchers and practitioners in the design, dissemination, and implementation of research. This necessitates the design of research and interventions to take into account the needs and contextual characteristics of practitioners and their practice settings in which research findings are intended to impact [23]. Bridging the gap and improving the alignment between the research and practice world will also require creating and sustaining an enabling environment for effective research engagement as well as dissemination, integration, and implementation of scientific evidence. Building capacity and organizational support structures will be essential as proposed by the push–pull–capacity model [42]. This model asserts that “for science to affect practice there must be a combination of the rationale for the science (pull), a demand for the science by practitioners (pull), and the delivery ability of the public health and healthcare systems (the capacity) [42].” However, based on our results and previous studies, [6,19] current gaps still exist. We found that only a third of all practitioners had adequate staffing and financial resources to implement research findings in their work. Similar organizational factors, including expectation by funding agencies and previous work in a practice or policy setting, were shown as significant determinants for dissemination efforts among researchers [13]. This suggests that strategies for capacity building, such as training, and strategies that build support structures at organizational level, such as staffing and funding, in both practice and research settings may contribute toward research translation. In building capacity, it will be critical to enhance equity by tailoring strategies based on specific needs of each setting and in consideration of contextual and social determinants and existing health disparities [27].

Our findings should be considered in light of a few limitations. We used self-report survey data which may be subject to recall bias or response bias. Depending on the roles of respondents within their organizations/clinics/hospitals, it is possible that respondents may not have had all the information about survey questions focused on the organizational settings. Given the low response rate (common in surveys of practitioners [43,44]), our findings may be subject to nonresponse bias. Those who responded may be different from those who did not respond regarding their perspectives on D4D. The low response rate affects the generalizability of the findings. We surveyed primary care physicians whose responses may not reflect experiences of other healthcare professionals. Given this study was the first to assess how clinical and public health practitioners in the USA learn about research evidence, future research is needed to examine D4D practice among other practitioner types within the healthcare professions as well as policymakers. Additionally, to gain a more comprehensive perspective on D4D and strategies to bridge the research–practice gap, it would have been helpful to have in-depth mixed or qualitative data to supplement our results. Despite these limitations, this is one of the few studies to examine how practitioners access and integrate research evidence, addressing a pertinent gap in our understanding of D4D. In addition, we were able to capture diverse experiences by surveying practitioners within public health and clinical settings.

## Conclusion

This study described how practitioners in the USA receive and use research evidence, which is important for researchers undertaking the practice of D4D to reach this audience. We found that there are differences in dissemination activities, research engagement, and organizational supports, among practitioners in contrast to researchers, in previous D4D studies. This provides important insights into where the persistent disconnect between the two worlds exist and provides the opportunity to identify points of intervention. The current study and existing literature suggests the need to identify and develop strategies and tools to more effectively D4D tailored to the needs of those adopting and implementing research evidence.

## Supporting information

Shato et al. supplementary material 1 Shato et al. supplementary material

Shato et al. supplementary material 2 Shato et al. supplementary material

## Supplementary material

The supplementary material for this article can be found at https://doi.org/10.1017/cts.2023.695.
