# Supplementary material for: Designing for dissemination among public health and clinical practitioners in the USA
Source: J Clin Transl Sci. 2023 Dec 14;8(1):e8. doi: 10.1017/cts.2023.695 (PMC10877519; doi:10.1017/cts.2023.695)
Supplement: Shato et al. supplementary material 1 — Shato et al. supplementary material [file S2059866123006957sup001.docx]

**Supplementary material 1 - Table S1:** Survey questions as informed by theoretical frameworks

| **Domain/**  **Construct** | **Question** | **Question No.** | **Theoretical framework** |
| --- | --- | --- | --- |
| Demographic characteristics | Please provide the zip code that corresponds to the city and state where you primarily work? (i.e. where your physical office is located)? | Q1 | N/A |
|  | In what type of setting do you work primarily (spend highest percent of your time)? | Q2 | N/A |
|  | What is the highest level of education that you have completed? | Q3 | N/A |
|  | What are the academic areas of your formal graduate degrees and/or fellowships? | Q4 | N/A |
|  | In what year did you receive your highest academic degree? | Q5 | N/A |
|  | I consider myself as more of a | Q6 | N/A |
| Awareness/ knowledge | Which of the following are your most trusted sources of information about new research findings? | Q7 | DOI |
|  | How do you most often get information about new research findings? | Q8 | DOI |
|  | If you were to receive findings from research related to your work focus area, how important would it be for the presentation of this research to have each of the following characteristics | Q9 | DOI |
|  | If you were to receive findings from research related to your work focus area, how important would it be for the presentation of this research to have each of the following characteristics | Q19 | DOI |
| Adoption | How often do you use research findings to do any of the following? | Q10 | K2A |
|  | What do you perceive as the biggest challenges, if any, to use of research findings in your work? | Q11 | K2A |
|  | Which of the following would encourage you to use research findings more often in your work? | Q12 | K2A |
|  | In my work setting, we are able to adapt to incorporating research findings in the work we do | Q13 | K2A |
| Implementation | In my work setting, we have adequate staffing to implement research findings in the work we do | Q14 | RE-AIM |
|  | In my work setting, we place a priority on promoting health equity in the work we do | Q15 | RE-AIM |
|  | In my work setting, we have adequate financial resources to implement research findings in the work we do | Q16 | RE-AIM |
|  | In my work setting, we track and monitor the use of research findings in the work we do | Q17 | RE-AIM |
|  | How important is the use of research findings in the work your organization/agency/clinic/hospital does? | Q18 | RE-AIM |
| Research engagement | In the past two years, what ways were you involved in research? | Q20 | N/A |
|  | How has your involvement in research changed since COVID-19? | Q21 | N/A |
| Recommendations for research dissemination | What are the best approaches for reaching people who do similar work to yours with the latest research findings? | Q22 | N/A |
|  | There is a considerable amount of inaccurate information (so called misinformation or disinformation) that is available to practitioners and the general public. What are a few things that could be done to overcome this presence of inaccurate information? | Q23 | N/A |
|  | Please use this space to leave any questions or comments about this survey. | Q24 | N/A |

DOI = Diffusion of Innovations; K2A = Knowledge to Action; RE-AIM = Reach Effectiveness Adoption Implementation Maintenance; N/A = Non-applicable
