# Supplementary material for: Designing for dissemination among public health and clinical practitioners in the USA
Source: J Clin Transl Sci. 2023 Dec 14;8(1):e8. doi: 10.1017/cts.2023.695 (PMC10877519; doi:10.1017/cts.2023.695)
Supplement: Shato et al. supplementary material 2 — Shato et al. supplementary material [file S2059866123006957sup002.docx]

Supplementary material 2 - Survey

Title

Designing for Dissemination among Public Health and Clinical Practitioners in the United States

Designing for Dissemination among Practitioners

Introduction

This project [team names and institutions provided] assesses the ways research findings are shared with practitioners across the United States. Your responses will help us to find ways to better communicate research findings to practitioners to support the use of scientific evidence into routine practice. We are asking clinical and public health practitioners to help us by completing this survey.   

The survey will take about **10 minutes of your time**. In appreciation for your help, we will provide a $50 gift card. At the end of the survey, you will be directed to a page giving details about the gift card, including an option to opt out. We will not collect your name or any identifying information about you in this survey. The full information about your rights as a participant in this study is offered in this [Consent information sheet](https://wustl.az1.qualtrics.com/CP/File.php?F=F_86bxwPCMmjzs6Ue). If you have any questions about the research study, please contact the study team [name and email provided].

Q1 Please provide the zip code that corresponds to the city and state where you primarily work? (i.e. where your physical office is located)?

- Zip code (if applicable): __________________________________________________
- Other: __________________________________________________

Q2 In what type of setting do you work primarily (spend highest percent of your time)?

- Local health department
- State health department
- Healthcare facility - inpatient (e.g. hospital, clinic)
- Healthcare facility - outpatient (e.g. hospital, clinic)
- University or school
- Community-based organization
- National organization. Please specify: __________________________________________________
- Other. Please specify: __________________________________________________

Q3 What is the highest level of education that you have completed?

- Some high school or less
- High school graduate; Trade/technical/vocational education beyond high school
- Some college
- Bachelor’s degree
- Master’s degree
- Doctoral degree (e.g., MD, PhD)
- Professional degree (e.g., LPN)
- Other. Please specify: __________________________________________________

| Page Break |  |
| --- | --- |

Q4 What are the academic areas of your formal graduate degrees and/or fellowships? [Please select all that apply]

- Behavioral Science (e.g. social work, psychology)
- Natural Sciences (e.g. biology, chemistry, physics)
- Medicine
- Nursing
- Public Health
- Policy
- Health Services Research
- Other. Please specify: __________________________________________________

Q5 In what year did you receive your highest academic degree?

▼ 1955 ... 2022

Q6 I consider myself as more of a

- Practitioner
- Researcher
- Both Practitioner and Researcher

Definition: For this survey, we define **research findings** as evidence produced through a rigorous process

|  |
| --- |

Q7 Which of the following are your most trusted sources of information about new research findings? [Please pick your top 3 choices, then rank-order your choices, where 1 is the most trusted]

| Please move and rank your top 3 choices into this box |
| --- |
| ______ National government agencies (e.g., CDC, FDA) |
| ______ State government agencies (e.g. state health departments) |
| ______ Local government agencies (e.g., local health departments) |
| ______ Researchers |
| ______ Professional associations (e.g., AMA, APHA) |
| ______ Advocacy organizations |
| ______ News media |
| ______ Social media |
| ______ Other. Please specify: |

|  |
| --- |

Q8 How do you most often get information about new research findings? [Please pick your top 3 choices, then rank-order your choices, where 1 is the most frequent]

| Please move and rank your top 3 choices into this box |
| --- |
| ______ Face to face/virtual meetings with stakeholders |
| ______ Email announcements/ alerts |
| ______ Reading academic journals |
| ______ Government reports |
| ______ Newsletters |
| ______ Policy briefs |
| ______ Social media (e.g. Twitter, Facebook) |
| ______ Podcasts |
| ______ Professional conferences |
| ______ Webinars |
| ______ Other. Please specify: |

Q9 If you were to receive findings from research related to your work focus area, how important would it be for the presentation of this research to have each of the following characteristics: [Please rate each statement below]

|  | Not at all important | Slightly important | Moderately important | Very important | Extremely important |
| --- | --- | --- | --- | --- | --- |
| It is delivered by someone I know and respect |  |  |  |  |  |
| It is relevant to the patients or populations we serve |  |  |  |  |  |
| It tells a story of how an issue affects the patients/populations we serve |  |  |  |  |  |
| It provides data on cost-effectiveness |  |  |  |  |  |
| It provides data on access/ insurance coverage |  |  |  |  |  |
| It presents practical advice about implementation |  |  |  |  |  |

Q10 How often do you use research findings to do any of the following?

|  | Never | Almost never | Sometimes | Almost every time | Every time | Not applicable |
| --- | --- | --- | --- | --- | --- | --- |
| Write a grant application |  |  |  |  |  |  |
| Plan or conduct a needs assessment |  |  |  |  |  |  |
| Develop a new program/policy/service |  |  |  |  |  |  |
| Modify an existing program/policy/service |  |  |  |  |  |  |
| Discontinue an existing program/ policy/service |  |  |  |  |  |  |
| Evaluate programs/policies/ services |  |  |  |  |  |  |
| Address the spread of inaccurate information |  |  |  |  |  |  |
| Promote health equity |  |  |  |  |  |  |

|  |
| --- |

Q11 What do you perceive as the biggest challenges, if any, to use of research findings in your work? [Please move and rank your top 3 choices into the box on the right, where 1 is the most important]

| Please move and rank your top 3 choices in this box |
| --- |
| ______ Lack of time to find and use research |
| ______ Lack of access to research |
| ______ Research not relevant to my work needs (including populations we serve) |
| ______ Research is not portrayed accurately in the media |
| ______ Lack of regular interaction with researchers |
| ______ Lack of brief summary of research findings |
| ______ Lack of training on how to access research findings |
| ______ Other. Please specify: |

Q12 Which of the following would encourage you to use research findings more often in your work? [Please move and rank your top 3 choices into the box on the right, where 1 is the most important]

| Please move and rank your top 3 choices in this box |
| --- |
| ______ Leaders or direct supervisors in my organization/agency placing a high priority on research |
| ______ Positive feedback or encouragement to use research |
| ______ Easy access to research findings or data sources |
| ______ Easy access to a summarized version of research findings |
| ______ An employee performance evaluation that supports the use of research findings |
| ______ Professional recognition for the use of research findings |
| ______ Making it part of job descriptions in my organization/agency |
| ______ Training to learn how to find and use research findings |
| ______ Other. Please specify: |
| Please indicate the extent to which you agree or disagree with each of the following statements below: |

|  | Strongly disagree | Disagree | Neither agree nor disagree | Agree | Strongly agree | Not sure/ Don't know |
| --- | --- | --- | --- | --- | --- | --- |
| Q13. In my work setting, we are able to adapt to incorporating research findings in the work we do |  |  |  |  |  |  |
| Q14. In my work setting, we have adequate staffing to implement research findings in the work we do |  |  |  |  |  |  |
| Q15. In my work setting, we place a priority on promoting health equity in the work we do |  |  |  |  |  |  |
| Q16. In my work setting, we have adequate financial resources to implement research findings in the work we do |  |  |  |  |  |  |
| Q17. In my work setting, we track and monitor the use of research findings in the work we do |  |  |  |  |  |  |

Q18 How important is the use of research findings in the work your organization/agency/clinic/hospital does?

- Not at all important
- Slightly important
- Moderately important
- Very important
- Extremely important

Q19 Does the organization/agency/hospital/clinic where you work have a designated individual or team responsible for finding and disseminating information on new research findings?

- Yes
- No
- Not sure

Q20 In the past two years, what ways were you involved in research? [Please check all that apply]

- Developing a research proposal
- Serving on an advisory committee
- Serving as a research participant (e.g., focus groups, interviews, user panels)
- Collecting data
- Providing testimonials or endorsements
- Interpreting data
- Writing up findings
- Disseminating findings through personal or professional networks
- I have not been involved in any research in the past 2 years
- Other. Please specify: __________________________________________________

Q21 How has your involvement in research changed since COVID-19?

- Decreased greatly
- Decreased slightly
- Stayed the same
- Increased slightly
- Increased greatly

Q22 What are the best approaches for reaching people who do similar work to yours with the latest research findings?

________________________________________________________________

________________________________________________________________

________________________________________________________________

________________________________________________________________

Q23 There is a considerable amount of inaccurate information (so called misinformation or disinformation) that is available to practitioners and the general public. What are a few things that could be done to overcome this presence of inaccurate information?

________________________________________________________________

________________________________________________________________

________________________________________________________________

________________________________________________________________

Q24 Please use this space to leave any questions or comments about this survey.

________________________________________________________________

________________________________________________________________

________________________________________________________________

________________________________________________________________

End of Block: Default Question Block
